# Sex, body size, and boldness shape the seasonal foraging habitat selection in southern elephant seals

**DOI:** 10.1002/ece3.8457

**Published:** 2022-01-26

**Authors:** Hassen Allegue, Christophe Guinet, Samantha C. Patrick, Mark A. Hindell, Clive R. McMahon, Denis Réale

**Affiliations:** ^1^ Département des Sciences Biologiques Université du Québec à Montréal Montréal QC Canada; ^2^ Centre d’études biologiques de Chizé CEBC CNRS Chizé France; ^3^ School of Environmental Sciences University of Liverpool Liverpool UK; ^4^ Institute for Marine and Antarctic Studies Battery Point TAS Australia; ^5^ Antarctic Climate and Ecosystems Cooperative Research Centre University of Tasmania Hobart TAS Australia; ^6^ Sydney Institute of Marine Science Sydney NSW Australia; ^7^ Department of Biological Sciences Macquarie University Sydney NSW Australia

**Keywords:** benefit–cost trade‐off, biologging, habitat selection, *Mirounga leonina*, personality

## Abstract

Selecting foraging habitat is a fundamental behavior in the life of organisms as it directly links resource acquisition to fitness. Differences in habitat selection among individuals may arise from several intrinsic and extrinsic factors, and yet, their interaction has been given little attention in the study of wild populations. We combine sex, body size, and boldness to explain individual differences in the seasonal foraging habitat selection of southern elephant seals (*Mirounga leonina*) from the Kerguelen Archipelago. We hypothesize that habitat selection is linked to the trade‐off between resource acquisition and risk, and that individuals differ in their position along this trade‐off because of differences in reproductive strategies, life stages, and metabolic requirements. Before the post‐molt foraging trip, we used a novel object approach test to quantify the boldness of 28 subadult and adult females and 42 subadult males and equipped them with data loggers to track their movements at sea. Subadult males selected neritic and oceanic habitats, whereas females mostly selected less productive oceanic habitats. Both sexes showed a seasonal shift from Antarctic habitats in the south in the summer to the free of ice subantarctic and subtropical habitats in the north in the winter. Males avoided oceanic habitats and selected more productive neritic and Antarctic habitats with body size mostly in the winter. Bolder males selected northern warmer waters in winter, while shyer ones selected the Kerguelen plateau and southern colder oceanic waters. Bolder females selected the Kerguelen plateau in the summer when prey profitability is assumed to be the highest. This study not only provides new insights into the spatiotemporal foraging ecology of elephant seals in relation to personality but also emphasizes the relevance of combining several intrinsic and extrinsic factors in understanding among‐individual variation in space use essential in wildlife management and conservation.

## Graphical abstract

Identifying factors driving individual differences in habitat selection is needed to understand population spatiotemporal dynamics essential in wildlife management and conservation. Here, we found that elephant seals in the Southern Ocean vary their seasonal foraging habitat selection with sex, body size, and boldness.

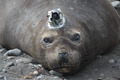

## 1 INTRODUCTION

Selecting where to forage is fundamental in organisms’ lives, and consequently, subject to a strong evolutionary pressure by natural selection (Pyke, 1984). Individuals within the same population, however, can differ in the habitats they select, which affects their performance and thus fitness (Bolnick et al., 2003). Habitat partitioning is thought to promote coexistence by reducing intra‐ or interspecific competition for resources (Araújo et al., 2011). Differences in habitat selection may emerge from ecological processes such as an ideal free or despotic distribution where individuals select more suitable habitats at a given time accordingly with environmental conditions (Fretwell & Lucas, 1969). And yet, increasing evidence shows an adaptive match between individual phenotypes and environmental conditions (Edelaar & Bolnick, 2019).

Individual habitat partitioning is explained by physiological and morphological constraints related to state variables such as sex (Istvanko et al., 2016), body size (Mittelbach, 1981), age (van den Hout et al., 2017), and polymorphism (Smith & Skúlason, 1996). However, these variables explain patially the among‐individual variation (Bolnick et al., 2003). Repeatable individual differences in behavioral traits through time or across contexts also known as personality (Réale et al., 2007) have emerged as a plausible descriptor to account for these unexplained differences in foraging behavior and habitat use (Spiegel et al., 2017). Personality has been found in almost every behavioral trait studied, for example, risk‐taking (Wilson et al., 1993) and exploration (Dingemanse et al., 2012), and has substantial implications for several ecological and evolutionary processes (Réale et al., 2010; Sih et al., 2012) including habitat choice. For example, fast‐exploring juvenile three‐spined sticklebacks (*Gasterosteus aculeatus*) use preferentially open habitats, whereas slow‐exploring individuals are found in covered habitats (Pearish et al., 2013). In urban great tits (*Parus major*), bolder individuals use areas with more cars and fewer pedestrians than shyer individuals (Sprau & Dingemanse, 2017). And bolder black‐browed albatrosses (*Thalassarche melanophris*) forage in shallow continental and coastal waters, whereas shyer individuals forage in deeper oceanic waters (Patrick & Weimerskirch, 2014).

The adaptive association between personality and habitat characteristics can emerge from four eco‐evolutionary mechanisms (Edelaar & Bolnick, 2019). Habitat‐specific pressures of natural selection can lead to local adaptations (Richardson et al., 2014), while animals can also adjust their environment differently according to their phenotypes to increase their fitness (Edelaar & Bolnick, 2019). Individuals may also select the environment they live in—for example, the matching habitat choice hypothesis for which individuals select the habitat that best suits their phenotypes (Edelaar et al., 2008). Finally, the correlation between personality and habitat could emerge from a plastic response of individuals to environmental conditions during ontogeny (Beaman et al., 2016) or from the habituation to environmental changes (Rankin et al., 2009).

Given that behavioral traits are moderately heritable (Stirling et al., 2002), and that personality differences can be targeted for selection (Smith & Blumstein, 2008), behavioral traits can drive the evolution of habitat selection. Personality‐habitat choice correlation should improve an individual's fitness by reducing stress and the costs associated with behavioral adjustments (Réale et al., 2007), which could arise from risk‐taking behaviors (Magnhagen & Borcherding, 2008), competing aptitude (Hansen & Closs, 2005), and social and anthropogenic tolerance (Martin & Réale, 2008). For example, differential foraging habitat in black‐browed albatrosses correlates with boldness which affects individuals’ fitness, and this effect varies in interaction between sex and the interannual variation in food availability (Patrick & Weimerskirch, 2014). Five mechanistic processes have been proposed to explain the link between behavioral traits, foraging behavior, and specialization: activity, fear and risk‐taking, social interactions, spatial movements, and internal physiological factors (Spiegel et al., 2017; Toscano et al., 2016). Thus, personality differences are likely to shape the movement and space use of individuals resulting in different biotic, abiotic, and social interactions (Chapman et al., 2011; Harrison et al., 2015; Spiegel et al., 2015). However, very little attention has been given to the effect personality has on habitat selection variation (Toscano et al., 2016) and was rarely combined into the same study framework with other state variables such as sex, age, or body size (but see Patrick & Weimerskirch, 2014; Yli‐Renko et al., 2018).

**FIGURE 1:**
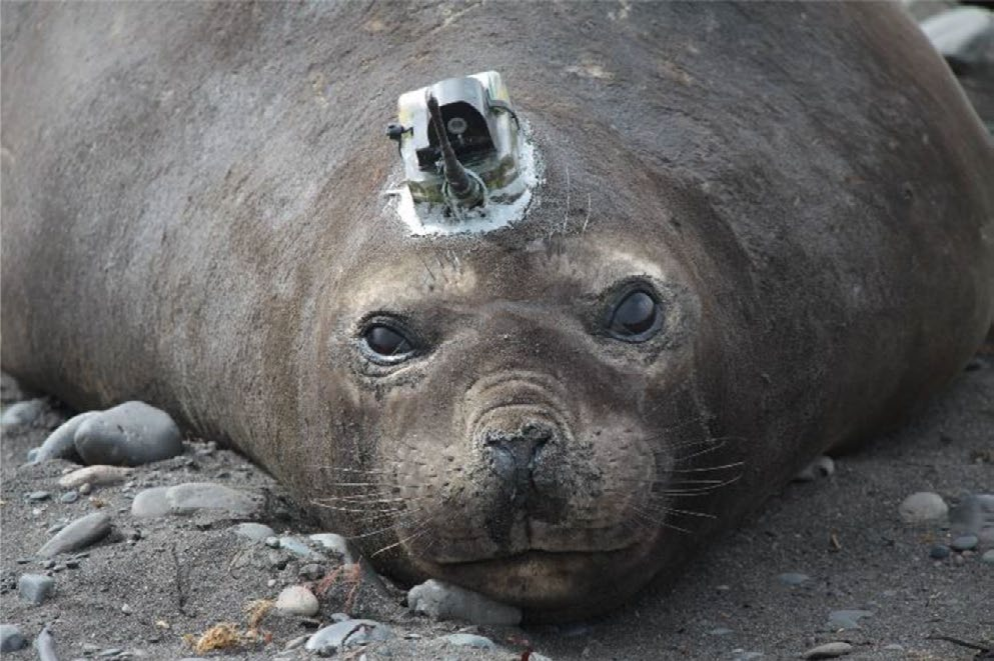
Photo of a female southern elephant seal equipped with a CTD Satellite Relay Data Logger

We investigate the role of personality, sex, and body size in shaping large‐scale foraging habitat selection in southern elephant seals (SES; *Mirounga leonina*; Figure 1) from the Courbet Peninsula in the Kerguelen Archipelago. SESs are the largest pinniped species inhabiting the Southern Ocean. Adults forage continuously at sea for up to 8 months and haul‐out ashore twice a year to breed and molt (Laws, 1956). Foraging performance in the capital breeding SES is crucial because seals must build large energy reserves, in the form of blubber, to support fasting during the long breeding and molting periods (1–2 months) on land (Laws, 1956). When foraging at sea, SESs range across most of the Southern Ocean, that is, from the subtropical front to the high Antarctic ice pack (Hindell et al., 2016). However, there is evidence for segregation in the seal core foraging areas such as shallow continental shelves and deep ocean water regions or inter‐frontal zones characterized by distinct oceanic characteristics and water masses (Guinet et al., 2014; Jonker & Bester, 1998). Early in life, SESs adopt specific foraging tactics by targeting particular habitats and diving depths, and consistently repeat these tactics over foraging seasons, leading to high individual foraging/diet specialization (Authier et al., 2012; Bradshaw et al., 2004; McIntyre et al., 2017).

Consistent individual differences in foraging habitat in SESs have been partly explained by sex and age classes (Bailleul, Authier, et al., 2010; Hindell et al., 2016, 2021). For example, subadult males from the Courbet Peninsula preferentially forage benthically on the Antarctic shelf and on the Kerguelen–Heard Plateau (Bailleul, Authier, et al., 2010; Dragon et al., 2010; Labrousse et al., 2015). In contrast, subadult and adult females forage mainly in deep pelagic ocean habitats generally along interfrontal zones or along marginal ice zones close to the Antarctic continent (Bailleul, Authier, et al., 2010; Labrousse et al., 2015). These distinct foraging habitats used by SESs (e.g., shelf vs. open ocean) vary in terms of resource profitability and degree of risk. For example, high‐Antarctic habitats support a higher productivity over the subantarctic habitats (Biuw et al., 2007; Park et al., 1998; Thums et al., 2011). However, the high seasonal and annual variability in both sea ice coverage and primary production in the Antarctic area results in higher variability in resource profitability (Constable et al., 2003; White & Peterson, 1996).

**FIGURE 2:**
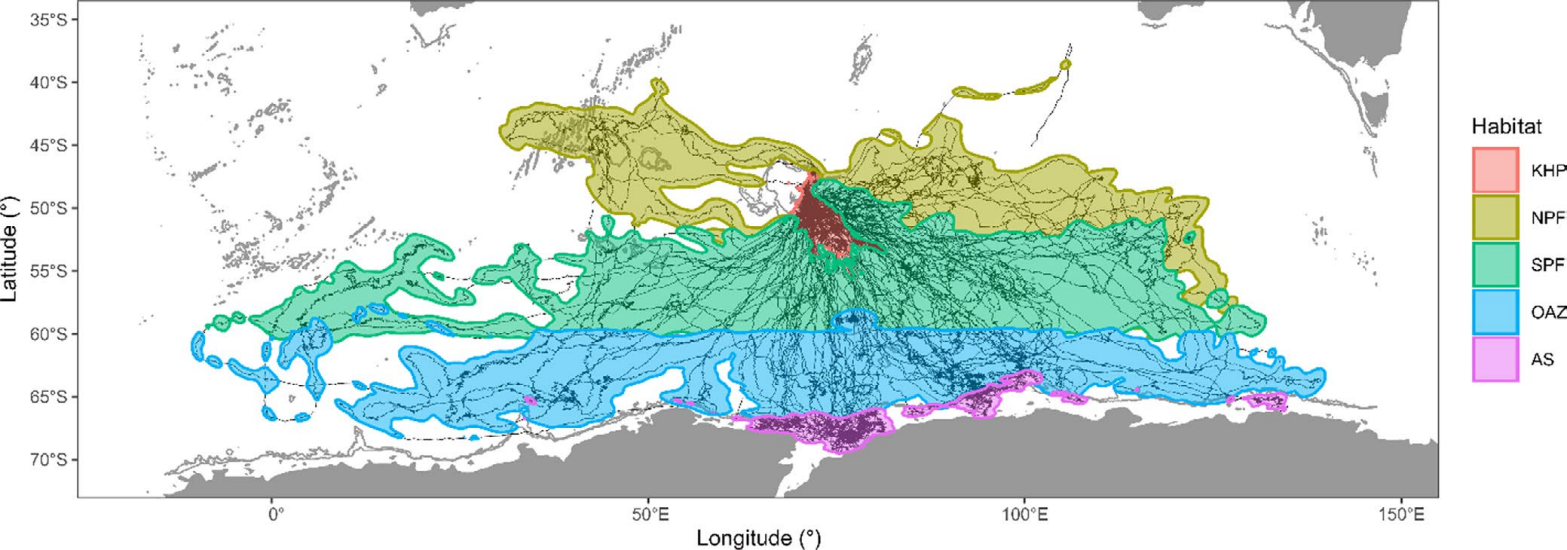
Overall spatial distribution of the habitat categories used by the southern elephant seals defined from the 99% contour of the kernel density. Habitats are the Kerguelen–Heard plateau (KHP), the North of Polar Front (NPF), the South of Polar Front (SPF), the Oceanic Antarctic Zone (OAZ), and the Antarctic Shelf (AS)

In this study, we identified five large‐scale foraging habitats used by SESs: two neritic habitats, that is, the Kerguelen–Heard Plateau (KHP) and the Antarctic Shelf (AS), and three oceanic habitats, that is, the North Polar Front (NPF), the South Polar Front (SPF), and the Oceanic Antarctic Zone (OAZ) (Figure 2). Based on our literature review, we built up our predictions in terms of the relative benefits and costs that SESs may face when foraging in these habitats (see Table 1 for a summary). Neritic habitats are more productive than oceanic habitats (Arrigo et al., 2008; Moore & Abbott, 2000). Within oceanic habitats, the Oceanic Antarctic Zone—with seasonal ice dynamics—has the highest productivity followed by the North Polar Front with warmer surface waters (Arrigo et al., 2008; Guinet et al., 2014; Moore & Abbott, 2000; Richard et al., 2016). In warmer waters, prey patches tend to be found in deeper depths in contrast with colder waters (Biuw et al., 2007; Guinet et al., 2014; McIntyre et al., 2011). In neritic habitats representing a smaller area and being more productive than oceanic habitats (Arrigo et al., 2008), we expect a higher intraspecific competition intensity. These habitats are also important foraging grounds for several other marine predators (e.g., pinnipeds, cetaceans, and seabirds), which increases interspecific competition (Hindell et al., 2011; Raymond et al., 2015; Siniff, 1991). Neritic habitats are expected to represent a higher risk of predation, for example, by sleeper sharks (*Somniosus antarcticus*) and killer whales (*Orcinus orca*) (Guinet et al., 1999; van den Hoff & Morrice, 2008; Walker et al., 1998). Southern habitats (i.e., OAZ and AS) are affected by the seasonal ice dynamics (Arrigo et al., 2008; Massom & Stammerjohn, 2010), which reduces their accessibility to air‐breathing marine predators and results in higher competition and susceptibility to predation. Neritic and ice‐covered habitats seem to exhibit higher inter‐ and intra‐annual variability in productivity mainly due to the ice pack dynamics (Arrigo et al., 2008; Massom & Stammerjohn, 2010). Productive areas in oceanic habitats tend to be predictable along interfrontal systems and (sub)mesoscale eddy structures (Bailleul et al., 2010; Cotté et al., 2015; Dragon et al., 2010).

**TABLE 1:** Descriptive summary of habitat relative benefits (i.e., productivity) and costs (i.e., competition, predation, ice cover density, and intra‐ and inter‐annual variability in productivity) used to develop our predictions and interpret our results

Habitat	Benefit	Cost				
	Productivity	Competition	Predation	Ice cover density	Intra‐ and interannual variability	Overall risk
Kerguelen–Heard Plateau (KHP)	High	High	High		Moderate	Moderate
North Polar Front (NPF)	Moderate	Low	Low		Low	Low
South Polar Front (SPF)	Low	Low	Low		Low	Low
Oceanic Antarctic Zone (OAZ)	Moderate	Moderate	Moderate	Moderate	High	Moderate
Antarctic Shelf (AS)	High	High	High	High	High	High
References	Arrigo et al. (2008), Biuw et al. (2007), Guinet et al. (2014), McIntyre et al. (2011), Moore and Abbott (2000), Richard et al. (2016) Thums et al. (2011)	Arrigo et al. (2008), Field, Bradshaw, Van Den Hoff, et al. (2007), Hindell et al. (2011), Raymond et al. (2015), Siniff (1991)	Campagna et al. (1995), Cox et al. (2020), Guinet et al. (1999), Henderson et al. (2020), van den Hoff and Morrice (2008), Walker et al. (1998)	Arrigo et al. (2008), Massom and Stammerjohn (2010)	Arrigo et al. (2008), Bost et al. (2009), Labrousse et al. (2017), Massom and Stammerjohn (2010)	

We hypothesize that boldness (i.e., risk‐taking/avoidance tendency) estimated during tests on land, in addition to sex and body size, contributes to explaining seasonal foraging habitat differences in SESs based on the trade‐off between food productivity and risks (i.e., competition, predation, ice‐cover density, or productivity variability; Table 1). We expect that (1) males forage more in shelf habitats (i.e., KHP and AS) whereas females favor oceanic pelagic habitats (i.e., NPF, SPF, and OAZ) due to differences in metabolic requirements and life‐history reproductive strategies (Hindell et al., 2016, 2021), (2) females—and not subadult males—that forage in the Antarctic area (i.e., OAZ and AS) will move northward as the ice pack expands during winter to avoid getting trapped there before the breeding season (Bailleul, Authier, et al., 2010; Labrousse et al., 2015), (3) males shift from oceanic to neritic habitats with body size while females predominantly forage in oceanic habitats (Bailleul, Authier, et al., 2010; Chaigne et al., 2013), and (4) individuals that forage in more profitable but riskier habitats, that is, the Kerguelen–Heard plateau (KHP) or the Antarctic continental shelf (AS), will be bolder than individuals that forage in less profitable but less risky habitats, that is, the subantarctic and the subtropical oceanic zones (Bonnot et al., 2018). We used the individual response intensity to a novel object approach test to quantify the boldness of individuals when on land and equipped them with a data logger to identify at‐sea habitat selection.

## 2 MATERIALS AND METHODS

### 2.1 Instrument deployment and data processing

In 2018 and 2019, we tracked 47 subadult male and 30 subadult and adult female southern elephant seals (SES) from the Kerguelen Islands (49.35°S, 70.22°E) during their post‐molting foraging trip at sea (January–September). The seals were equipped with CTD Satellite Relay Data Loggers (CTD‐SRDL, Sea Marine Research Unit, University of St Andrews) which transmitted Argos locations, salinity and temperature (S/T) profiles, and dive profiles summarized onboard into five time–depth segments using the broken‐stick algorithm (for more technical details, see Boehme et al., 2009). We captured seals with a canvas head‐bag and sedated them with a 1:1 combination of Tiletamine and Zolazepam (Zoletil 100) injected intravenously (McMahon et al., 2000). The body length of seals was measured from the nose tip to the tail tip when laying flat, which we used as an indicator of body size. Loggers were glued to seal heads with quick‐setting epoxy (Araldite AW 2101, Ciba) (McMahon et al., 2008). Individual tracks were estimated by filtering observed locations with a correlated random walk state‐space model with a 6‐h time step that accounts for error in the Argos system (R package foieGras) (Jonsen et al., 2020). Dive and S/T profiles were not recorded and transmitted at the same time, and thus, we assigned to each dive profile the median S/T profile within a range of 12 h before and after the dive time. Each logger transmitted on average 3.2 ± 1.2 S/T profiles per day. When no S/T profiles were transmitted from the tags, we used S/T profiles from the WOA18 (World Ocean Atlas) database provided by NOAA (www.nodc.noaa.gov). We included in our analysis foraging trips that lasted at least 45 days whether data transmission stopped before the seal returned on land or not. This threshold was used as it represented the duration of the shortest round trip. Round trips at sea were considered completed when the seal hauled out for at least a week on Kerguelen or Heard Islands. All data manipulation and analyses were done in R 4.03 (R Core Team, 2020).

### 2.2 Habitat delimitation and characteristics

Habitat categories were defined based on previously documented large‐scale foraging areas of SESs (Bailleul, Authier, et al., 2010; Guinet et al., 2014; Labrousse et al., 2015) (Figure 2 and Figure S5). We first defined the Kerguelen–Heard Plateau (KHP) delimited from 56° to 45.5°S and 61° to 83°E within a bathymetry shallower than 2000 m. Second, we separated the oceanic area into three areas: the North of the Polar Front (NPF) with a temperature at 200 m higher than 2°C, the South of the Polar Front (SPF) with a temperature at 200 m lower than 2°C (Orsi et al., 1995), and the Antarctic zone below a latitude of 60°S or at the maximum ice pack extend during the year. The Antarctic zone was further split into two areas: The Oceanic Antarctic Zone (OAZ) with a bathymetry deeper than 2000 m and the Antarctic Shelf (AS) with a bathymetry shallower than 2000 m. We used the GEBCO bathymetry dataset at 15 arc‐second resolution (www.gebco.net) and ice concentration was extracted from the sea ice remote‐sensing dataset of the University of Bremen at 6.25 km resolution (Spreen et al., 2008).

We tested whether interindividual variation in habitat selection is driven by the tendency of individuals to take or avoid risks when foraging. Thus, based on our literature review, we estimated the relative benefits (i.e., productivity) and costs (i.e., the intensity of the competition and predation, the pack ice density, and the inter‐ and intra‐annual variability in productivity) associated with each of the five foraging habitats (Table 1). We then summarized the relative risk level of each habitat based on all the cost variables described in Table 1. It is important here to keep in mind that the habitat risk levels were never explicitly tested for SESs, but rather defined according to the information found on these habitats in the literature.

### 2.3 Novel object approach test

To quantify individual boldness, we conducted a novel object approach test on every individual (Patrick & Weimerskirch, 2014). We used as a novel object an inflatable pink toy cow (dimensions ~45 × 25 × 25 cm; Farm Hoppers^®^) attached to the end of a 5‐m pole. Each test was recorded with a goPro camera attached on top of the toy cow and was conducted as follows: (1) A seal was chosen when the area in front or beside it was free to access, (2) a human (the tester), wearing dark blue or black clothing and starting at approximately 10 m, pushed the novel object along the ground at a regular pace in front of the focal seal until it reached ca. 1 m, and (3) waited for at least 30 s while recording the behavioral response of the focal seal. If the seal moved, the tester adjusted the distance between the object and the seal accordingly to preserve, as much as possible, the ca. 1 m distance between them. The test lasted 35 s, which included the last 5 s of the approach phase plus the first 30 s of the waiting phase. Repeated tests on the same individuals were separated by at least 24 h. We ran between two and five tests on 13 individuals and one test on 63 individuals for a total of 101 tests (Figure S2).

Behavioral test recordings were analyzed with the software BORIS v7.4.14 (Friard & Gamba, 2016). We extracted the proportion of time of several behavioral responses: (1) rising head, (2) standing on fore flippers, (3) opening mouth, (4) vocalization, (5) moving backward (i.e., retreating), and (6) attacking the object by head strokes or by moving forward (i.e., charging). Rising head and standing were split into two levels: “*low”* when the seal's snout was parallel to the floor and the head was approximately at the body height, and “*high”* when the seal's snout pointed out toward the sky and the head was higher than the body height. Some behavioral responses were mutually inclusive such as if a seal is standing, its head is also lifted, and if a seal is vocalizing, its mouth is also open.

We used a principal component analysis (PCA) on all normalized behavioral response variables (i.e., zero mean and unit variance) and used the first principal component (PC1) to reduce the behavioral response into one value specific to each test. A unique PC1 score was estimated for each individual as the average best linear unbiased predictor from 1000 simulations of a univariate linear mixed‐effect model (Dingemanse et al., 2019). The model accounted for the study year (i.e., 2018 or 2019), the number of conspecifics within 3 m radius from the focal seal, the position of the focal seal (i.e., straight or not), whether the seal was in a huddling group (i.e., no huddling, edge, or inside the group), the human approach direction (i.e., front or side), the number of previous tests, and the number of captures as fixed effects. Behavioral tests were conducted either before or after seals were captured for logger deployment. Furthermore, as part of another monitoring program some seals were captured twice, at the beginning and at the end of the molting period. To control for potential effects of the capture on SESs behavioral responses toward humans, we thus included the number of captures in the model. The final model only included predictors of the most parsimonious model based on the lowest Akaike information criterion corrected for small sample sizes (AIC_c_). We also included the seal and the tester identifier, and the date as random effects to account for repeated measurements and other daily environmental variation that we did not collect (e.g., weather).

The adjusted repeatability, defined as the proportion of the total variance attributed to differences among individuals after accounting for confounding factors, was calculated from the mixed‐effect model fitted to the PC1 axis using the R package rptR (Nakagawa & Schielzeth, 2010). We used parametric bootstrapping (1000 bootstraps) to estimate the 95% confidence interval and all individuals including those with one trial were used to improve the power of the repeatability estimate (Martin, Nussey, et al., 2011). The repeatability score is used as an indicator of the consistency of the individual behavioral response over multiple tests.

### 2.4 Statistical analyses

To determine which intrinsic parameters explain the variability of individual seals in habitat selection, we used a resource selection function approach (Johnson et al., 2006) with the following model equation:

$$w\left(hab_{\mathrm{ijk}}\right)=\exp\left(\beta X+\gamma_{\mathrm{ij}}^{\left(trip\right)}hab_{\mathrm{ijk}}+\gamma_{\mathrm{ik}}^{\left(seal\right)}hab_{\mathrm{ijk}}\right)$$

where $w\left(hab_{\mathrm{ijk}}\right)$ is the relative probability of selecting habitat (*i*) at the trip (*j*) by the seal (*k*), ***β*** is the vector of the coefficients in response to a set of predictors (**X**), $\gamma_{\mathrm{ij}}^{\left(trip\right)}$ is the trip‐specific (*j*) random intercept value for habitat (*i*), and $\gamma_{\mathrm{ik}}^{\left(seal\right)}$ is the seal‐specific (*k*) random intercept value for habitat (*i*). The matrix of predictors (**X**) included two interaction terms between habitat (i.e., KHP, NPF, SPF, OAZ, and AS), sex, and season with either body length or PC1. The post‐molt foraging period ranging from January to August was split into two distinct seasons: *summer* (January–April) and *winter* (May–August). All continuous predictors were normalized (i.e., zero mean and unit variance). We also modeled the correlation in the habitat selection variance within trips.

Model parameters were estimated by fitting a binomial logistic linear mixed‐effect model for which habitats used each day (coded as “1”) are compared to habitats available (coded as “0”) to seals starting their foraging trip from the Kerguelen Islands. If more than one habitat was used during the same day, only the one with the highest number of occurrences was kept. The number of availability values for each habitat within the same seal trip was kept the same as the number of true daily observations. Habitat availability values were generated according to the proportion of occurrence of each habitat in simulated pseudo‐tracks. We adopted a simulation‐based approach for which we generated 100 pseudo‐tracks per individual by fitting a first‐order vector autoregressive model on the true tracks randomly selected from a subset of all seal tracks (availability R package) (Hindell et al., 2020). The subset of the true tracks included tracks with intermediate maximum distance from the colony representing general movement patterns in the whole area. This subset of tracks was defined from a hierarchical clustering method with three clusters on the maximum distance from the colony using the Ward's metric and the Euclidean distance. This method was used to avoid any bias related to extreme and unbalanced movement patterns among the seals we equipped. Therefore, simulated pseudo‐tracks had realistic movement characteristics (i.e., step length and turning angle) while random and independent of environmental factors, and were minimally biased by the behavior of the seals we equipped (Figure S4).

The model was fitted following a Bayesian approach using the brms R package (Bürkner, 2017). We used four chains with 10,000 iterations from which 4000 for warmup, and 99% average acceptance probability. The model priors were chosen based on visual inspection of the prior predictive distributions: a normal distribution (mean = 0 and SD = 1) for all ***β*** parameters, a Cauchy distribution (location = 0 and scale = 2) for the variance of the random parameters, and the LKJ distribution (shape = 1) for correlation parameters. We randomly resampled 50% of the data to reduce the effect of the spatiotemporal autocorrelation.

## 3 RESULTS

After filtering the raw dataset, we analyzed the data of 42 subadult males and 28 subadult and adult females. Males measured 2.29 ± 0.20 m (min: 1.92, max: 2.62 m) and females 2.30 ± 0.17 m (min: 1.95, max: 2.61 m; Figure S3). Males and females did not differ in body length (Welch *t*‐test: df = 64.08, *t* = 0.28, *p* = .778). Foraging trips at sea lasted on average 113 ± 39 days for males and 190 ± 60 days for females (Welch *t*‐test: df = 42.14, *t* = 6.58, *p* = <.001), and the maximum distance from the Kerguelen Islands of foraging trips was on average 1770 ± 1077 km for males and 2789 ± 1137 km for females (Welch *t*‐test: df = 55.13, *t* = 4.22, *p* = <.001).

### 3.1 Boldness

The first axis of the PCA accounted for 41.4% of the total variance in the seal behavioral response to the novel object approach test (Figure S1 and Table S1). Loadings of the PC1 were negative for all behaviors with the lowest values associated with head elevation, standing, and moving away (Table S1). We interpreted PC1 as the intensity of the seal stress response to the test which we refer to as *boldness*. Boldness ranged along the continuum between “*shy”* individuals that responded intensively to the test and “*bold”* individuals that had none or very low response (Bubac et al., 2018). Individual seals showed an adjusted repeatability of 0.28 ± SE: 0.15 (confidence interval: [0.03–0.64]; *p*: .021) in boldness. The final model for boldness (i.e., after model selection based on the lowest AICc) included the position of the seal, the number of captures, and the year as fixed effects (Table S2).

### 3.2 Relative habitat selection

#### 3.2.1 Sex

**FIGURE 3:**
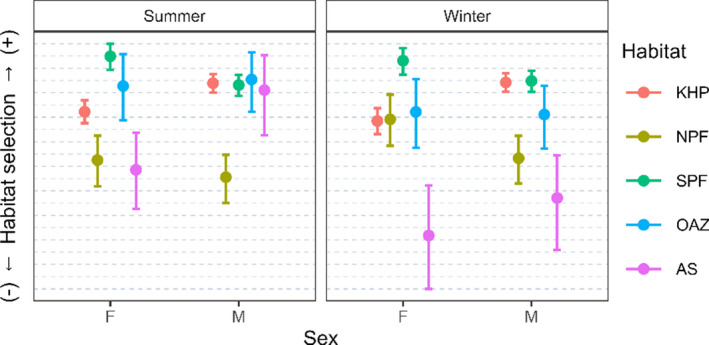
Predicted log relative foraging habitat selection (with 95% credible intervals) in each habitat category per sex (M = male and F = female) and season (summer and winter). Habitats are the Kerguelen–Heard plateau (KHP), the North of Polar Front (NPF), the South of Polar Front (SPF), the Oceanic Antarctic Zone (OAZ), and the Antarctic Shelf (AS). Values of the y‐axis are not shown as they are at a relative scale, and therefore, do not have any relevant meaning

Males selected neritic habitats (the Kerguelen–Heard plateau and the Antarctic Shelf) whereas females preferentially selected oceanic habitats (the North and South Polar Front) for both seasons (Figure 3 and Table 2). However, sexes did not differ in their selection of the North Polar Front in summer, the Antarctic Shelf in winter, and the Oceanic Antarctic Zone in summer and winter. Both sexes switched foraging habitats from predominantly southern habitats in the summer to more northern habitats in the winter (Figure 3 and Table 3). Males shifted from the Antarctic Shelf and the Oceanic Antarctic Zone in the summer to the North Polar Front in the winter, while females shifted from the Antarctic Shelf, the Oceanic Antarctic Zone, the South Polar Front, and the Kerguelen–Heard Plateau in the summer to the North Polar Front in the winter.

**TABLE 2:** Prediction output of sex differences in habitat selection within seasons (i.e., summer and winter)

Season	Habitat	Estimate of male–female (SE) [CI]
Summer	KHP	**0.95 (0.22) [0.52, 1.38]**
	NPF	−0.54 (0.50) [−1.54, 0.44]
	SPF	**−0.96 (0.26) [−1.46, −0.44]**
	OAZ	0.24 (0.58) [−0.91, 1.38]
	AS	**2.61 (0.70) [1.20, 3.94]**
Winter	KHP	**1.27 (0.24) [0.80, 1.75]**
	NPF	**−1.25 (0.51) [−2.26, −0.27]**
	SPF	**−0.65 (0.26) [−1.16, −0.13]**
	OAZ	−0.11 (0.61) [−1.31, 1.08]
	AS	1.27 (0.93) [−0.58, 3.07]

Note

Estimates are the posterior mean effect differences between males and females and are presented with standard errors (SE) and 95% credible intervals (CI). Habitats are the Kerguelen–Heard plateau (KHP), the North of Polar Front (NPF), the South of Polar Front (SPF), the Oceanic Antarctic Zone (OAZ), and the Antarctic Shelf (AS). Bolded estimates do not include zero in their credible interval.

**TABLE 3:** Prediction output of season (i.e., summer and winter) differences in habitat selection for each sex

Sex	Habitat	Estimate of winter–summer (SE) [CI]
Male	KHP	0.04 (0.09) [−0.13, 0.21]
	NPF	**0.60 (0.16) [0.28, 0.91]**
	SPF	0.15 (0.11) [−0.06, 0.36]
	OAZ	**−1.09 (0.20) [−1.48, −0.70]**
	AS	**−3.39 (0.40) [−4.21, −2.64]**
Female	KHP	*−0.27 (0.15) [−0.57, 0.02]*
	NPF	**1.31 (0.11) [1.09, 1.53]**
	SPF	*−0.16 (0.09) [−0.34, 0.02]*
	OAZ	**−0.73 (0.21) [−1.15, −0.31]**
	AS	**−2.05 (0.62) [−3.29, −0.86]**

Note

Estimates are the posterior mean effect differences between winter and summer and are presented with standard errors (SE) and 95% credible intervals (CI). Habitats are the Kerguelen–Heard plateau (KHP), the North of Polar Front (NPF), the South of Polar Front (SPF), the Oceanic Antarctic Zone (OAZ), and the Antarctic Shelf (AS). Bolded estimates do not include zero in their credible interval and italic ones do include zero but it is within 0.05 from one of the interval ends.

#### 3.2.2 Body size

**FIGURE 4:**
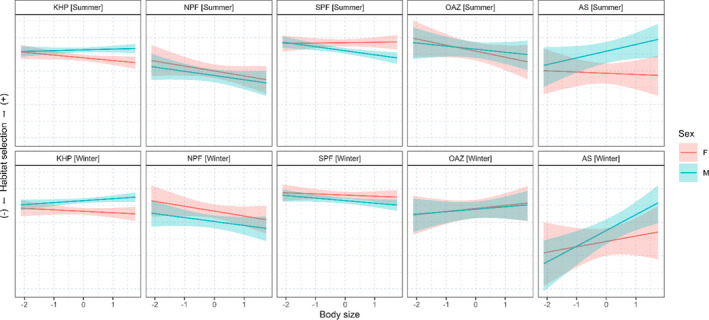
Predicted log relative foraging habitat selection for each habitat category as a function of body size (*z*‐score). Habitats are the Kerguelen–Heard plateau (KHP), the North of Polar Front (NPF), the South of Polar Front (SPF), the Oceanic Antarctic Zone (OAZ), and the Antarctic Shelf (AS). Values of the y‐axis are not shown as they are at a relative scale, and therefore, do not have any relevant meaning

Males tended to avoid oceanic habitats and select neritic habitats with body size, and this effect was more apparent in the winter than in the summer (Figure 4 and Table 4). In females, no clear pattern was found between habitat selection and body size (Figure 4 and Table 4), but we found a slight decrease in the selection of the Kerguelen–Heard Plateau with body size in summer. In both sexes, the effect of body size on the selection of the Oceanic Antarctic Zone varied seasonally and was stronger in the winter than in the summer (male: winter–summer 0.66 ± SE: 0.22 [credible interval: 0.24, 1.09]; female: 1.09 ± 0.23 [0.65, 1.55]).

**TABLE 4:** Estimates of the habitat selection model (logistic model) for which the response variable was coded as “1” for observed habitat use and “0” for habitat availability

Fixed effects	Habitat‐specific log‐odd ratio (SE) [95% CI]				
	KHP	NPF	SPF	OAZ	AS
Summer					
Female					
Intercept	**−2.43 (0.19)** **[−2.79, −2.06]**	**−4.00 (0.48)** **[−4.85, −3.22]**	**−0.61 (0.31)** **[−1.05, −0.21]**	**−1.63 (0.57)** **[−2.74, −0.61]**	**−4.28 (0.64)** **[−5.54, −3.09]**
Length	*−0.33 (0.19)* *[−0.70, 0.04* *]*	−0.59 (0.47) [−1.40, 0.21]	0.05 (0.30) [−0.37, 0.47]	−0.73 (0.52) [−1.68, 0.24]	−0.15 (0.61) [−1.28, 1.04]
Boldness	*0.31 (0.18)* *[−0.05, 0.66]*	0.19 (0.45) [−0.54, 0.93]	0.03 (0.28) [−0.34, 0.41]	0.14 (0.51) [−0.78, 1.08]	0.38 (0.61) [−0.76, 1.55]
Male					
Intercept	**−1.47 (0.22)** **[−1.77, −1.18]**	**−4.54 (0.57)** **[−5.36, −3.81]**	**−1.57 (0.36)** **[−1.93, −1.23]**	**−1.38 (0.61)** **[−2.42, −0.50]**	**−1.63 (0.71)** **[−3.08, −0.51]**
Length	0.09 (0.21) [−0.17, 0.36]	−0.50 (0.56) [−1.19, 0.18]	**−0.49 (0.35)** **[−0.80, −0.18]**	−0.37 (0.59) [−1.18, 0.43]	0.80 (0.66) [−0.11, 1.75]
Boldness	0.06 (0.22) [−0.26, 0.37]	0.02 (0.58) [−0.80, 0.84]	*−0.32 (0.35)* *[−0.70, 0.04]*	−0.25 (0.61) [−1.16, 0.63]	0.08 (0.68) [−0.94, 1.12]
Winter					
Female					
Intercept	**−2.70 (0.15)** **[−3.11, −2.28]**	**−2.70 (0.19)** **[−3.54, −1.90]**	**−0.77 (0.17)** **[−1.22, −0.36]**	**−2.36 (0.25)** **[−3.51, −1.32]**	**−6.31 (0.61)** **[−8.01, −4.73]**
Length	−0.17 (0.16) [−0.58, 0.24]	−0.59 (0.20) [−1.40, 0.22]	−0.13 (0.19) [−0.57, 0.29]	0.36 (0.27) [−0.63, 1.37]	0.64 (0.63) [−0.96, 2.25]
Boldness	*−0.38 (0.14)* *[−0.76, 0.01]*	0.48 (0.17) [−0.24, 1.22]	0.09 (0.16) [−0.29, 0.46]	−0.32 (0.27) [−1.29, 0.66]	−0.16 (0.60) [−1.67, 1.45]
Male					
Intercept	**−1.43 (0.17)** **[−1.73, −1.13]**	**−3.95 (0.25)** **[−4.76, −3.21]**	**−1.42 (0.22)** **[−1.77, −1.09]**	**−2.46 (0.32)** **[−3.55, −1.55]**	**−5.02 (0.65)** **[−6.67, −3.67]**
Length	*0.23 (0.18)* *[−0.04, 0.51]*	−0.47 (0.26) [−1.15, 0.20]	*−0.30 (0.23)* *[−0.62, 0.01]*	0.29 (0.34) [−0.55, 1.13]	**1.88 (0.66)** **[0.77, 3.05]**
Boldness	*−0.31 (0.17)* *[−0.64, 0.01]*	**0.82 (0.27)** **[0.01, 1.65]**	**−0.50 (0.22)** **[−0.88, −0.13]**	0.25 (0.33) [−0.67, 1.17]	0.75 (0.65) [−0.43, 1.99]
Among‐individual variance	*0.12 (0.08)* *[0.00, 0.30]*	**0.94 (0.50)** **[0.06, 1.89]**	**0.23 (0.16)** **[0.01, 0.59]**	**0.56 (0.41)** **[0.02, 1.52]**	**0.52 (0.40)** **[0.02, 1.49]**

Note

The sex (female or male), the season (summer or winter), the body length, and the boldness were included as predictors. All continuous predictors were standardized (i.e., zero mean and unit variance). Effect size estimates are presented as the mean log odds ratios. Note that effect sizes have been measured for each combination of the categorical variables (i.e., season and sex), and thus do not depend on level reference coding. However, effect sizes of resource selection functions should be interpreted relative to each other. Standard errors (SE) and 95% credible intervals [CI] are reported for each estimate. Bolded estimates do not include zero in the credible interval and italic ones do include zero but it is within 0.05 from one of the interval ends. Habitats are the Kerguelen–Heard plateau (KHP), the North of the Polar Front (NPF), the South of the Polar Front (SPF), the Oceanic Antarctic Zone (OAZ), and the Antarctic Shelf (AS).

#### 3.2.3 Boldness

**FIGURE 5:**
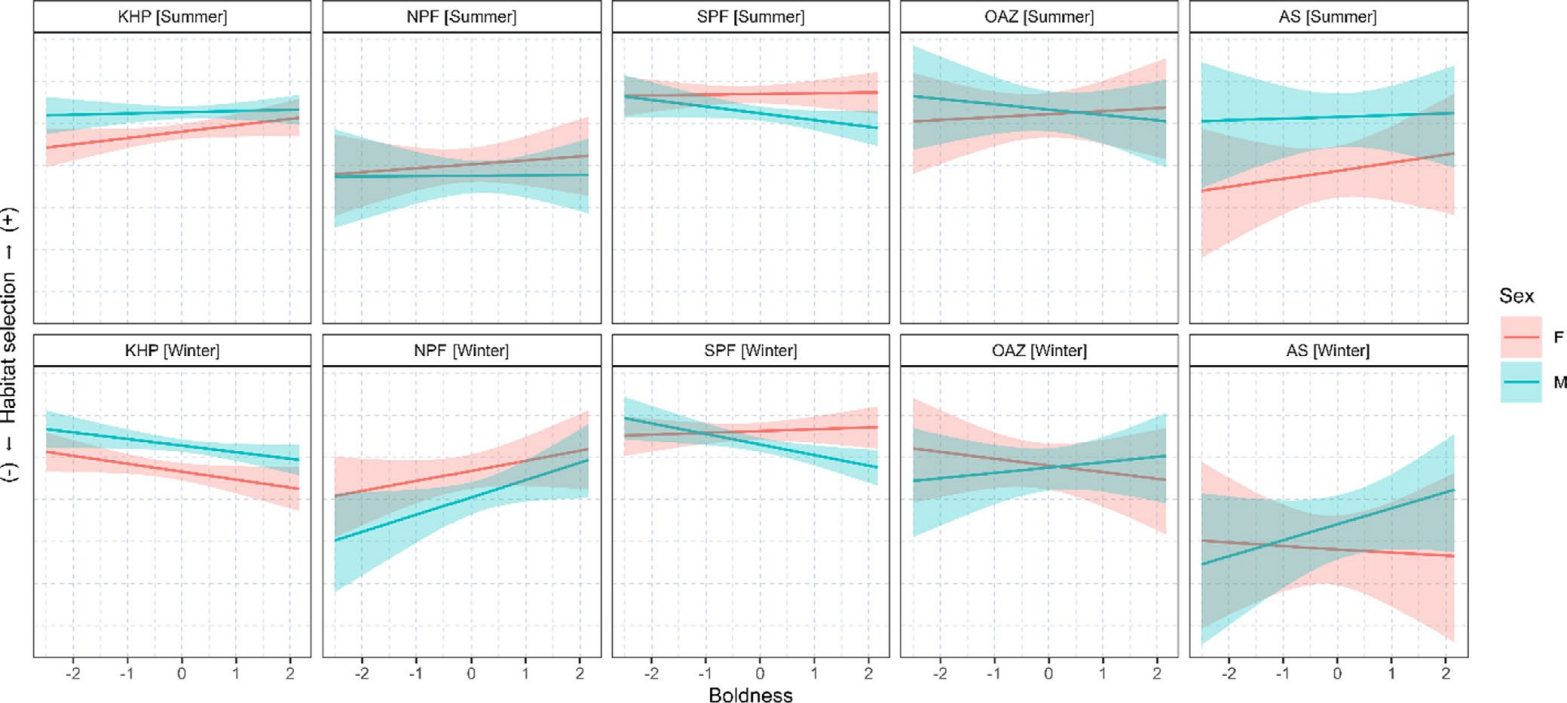
Predicted log relative foraging habitat selection as a function of boldness (*z*‐score). Habitats are the Kerguelen–Heard plateau (KHP), the North of Polar Front (NPF), the South of Polar Front (SPF), the Oceanic Antarctic Zone (OAZ), and the Antarctic Shelf (AS). Values of the *y*‐axis are not shown as they are at a relative scale, and therefore, do not have any relevant meaning

We found that boldness affected habitat selection in males mainly in winter (Figure 5 and Table 4). Bolder males avoided the Kerguelen–Heard Plateau and the South Polar Front and selected the North Polar Front in winter with boldness. In summer, we found that males only avoided the South Polar Front. Additionally, we found a stronger positive effect of boldness on the selection of both Antarctic habitats in winter than in summer (OAZ: winter–summer 0.51 ± 0.20 [0.13, 0.90]; AS: 0.68 ± 0.39 [−0.07, 1.46]). In females, boldness did not strongly affect habitat selection (Figure 5 and Table 4). However, we found a negative effect of boldness on the selection of the Kerguelen–Heard Plateau in the winter in contrast to summer.

## 4 DISCUSSION

We found that SESs varied in foraging habitat selection according to sex, body size, and boldness, which varied between summer and winter. We show that this interindividual variability may be partially driven by the trade‐off between the benefits and costs associated with each habitat, and that individuals may be positioned along this trade‐off axis according to differences in energy requirements, reproductive strategy, life‐history stage, and predation risk.

### 4.1 Sex

Male seals selected more productive but riskier neritic habitats whereas females mainly selected safer but less productive oceanic habitats. Such sex‐specific differences in foraging habitats were largely documented in most SES populations all around the Southern Ocean which is likely explained by differences in energy constraints and life‐history reproductive strategies (Bailleul, Authier, et al., 2010; Hindell et al., 1991, 2021; McConnell & Fedak, 1996). Females breed almost annually, starting at an early age (~3–5 years old; McMahon et al., 2008), whereas males, under high intra‐sexual pressure, start breeding marginally at ca. 6 years of age but reach their prime breeding years between the age of 9 and 12 (Laws, 1956). Breeding females must increase body fat reserves to support the high cost of the upcoming annual lactation (Fedak et al., 1996), while non‐breeding subadult males allocate most of their energy in growth to increase body size (Field et al., 2007)—the main trait affecting breeding success (Modig, 1996). This dichotomy in life‐history strategies between sexes may explain the divergence in foraging habitats which may result in sex‐specific long‐term fitness optima.

Neritic habitats are the most productive habitats in which SESs of both sexes build fat contents more efficiently compared to oceanic habitats (Authier et al., 2012; Schick et al., 2013; Thums et al., 2011). Males foraging there would then profit preferentially to maximize growth rate—essential for late‐life breeding success given that only ca. 4% of males hold harems (Le Boeuf & Peterson, 1969). Although very productive, these habitats may also be costly especially for females which could explain why they tend to avoid them. Firstly, neritic habitats support higher intra‐ and intersexual competition and predation risk than oceanic habitats (Table 1). Secondly, interannual variability in productivity in neritic habitats is higher than in oceanic habitats (Arrigo et al., 2008). Males may benefit more from foraging in neritic habitats buffering for this variability in productivity as they must maximize growth rate at the lifespan scale and not annually, whereas it may be highly costly for females as they give birth to only one pup per year. Females may hence adopt a bet‐hedging strategy for which they select less productive but safer habitats to secure annual reproductive success along their life (Simons, 2011). And third, the intra‐ and interannual productivity and accessibility of the Antarctic shelf are highly influenced by ice dynamics (Labrousse et al., 2015; Massom & Stammerjohn, 2010).

### 4.2 Season

Females move away from the Antarctic shelf as the ice edge expands while non‐breeding males stay in the ice pack foraging in coastal polynyas (Bailleul et al., 2007; Labrousse et al., 2017, 2018). Our results confirm this behavior in females that avoid the risk of getting trapped in the thick ice pack to return to Kerguelen Islands for breeding. A similar trend of moving northward as the pack ice grew was also observed in adult males when approaching reproductive age (Biuw et al., 2010). We found that subadult males avoided ice‐covered habitats in the winter similar to females. This could be explained by the young age (i.e., small body size) of the seals that we equipped compared to previous studies in the Kerguelen Islands (Bailleul et al., 2007; Labrousse et al., 2017). Juvenile males tend to return ashore in mid‐winter to rest (Hindell & Burton, 1988; Hindell et al., 2021). This conclusion is also supported by the positive relationship that we found between the body size of the males and the selection of the Antarctic Shelf, especially in the winter.

### 4.3 Body size

We found that males tended to shift from oceanic to neritic habitats with body size which is consistent with previous studies that considered body size as a proxy of age. For example, stable isotope analyses showed that subadult SES males, and not females, from Kerguelen shifted from oceanic and polar frontal waters to neritic and Antarctic waters at 3–4 years of age (Bailleul, Authier, et al., 2010; Chaigne et al., 2013), corresponding to a body length of ca. 2.1–2.5 m (Bell et al., 2005; McLaren, 1993). After this age threshold, males seem to specialize in foraging either on the Kerguelen or the Antarctic plateau and stay faithful to that habitat while increasing gradually trophic level with age (Authier, Bentaleb, et al., 2012; Martin, Bentaleb, et al., 2011). We found no changes in female habitat selection with body size. However, females avoided the Kerguelen–Heard Plateau in the summer and increased the selection of the Oceanic Antarctic Zone in the winter with body size. Younger, and therefore smaller, SESs tend to forage closer to their haul‐out island (Field et al., 2007), which may explain the first result. However, several studies showed that naïve pups and 1‐ to 4‐year‐old juvenile male and female SESs foraged mainly in oceanic waters (Field et al., 2005; McConnell et al., 2002) and those that stayed on the Kerguelen plateau were less successful in increasing their body condition (Orgeret et al., 2018). Also, the ability to forage in ice‐covered areas may be related to body size. For example, larger females in the Antarctic Peninsula foraged in areas with higher ice concentrations than smaller females (Muelbert et al., 2013). In this study, body length ranged between ca. 1.9 and 2.6 m corresponding to an age range of ca. 1–5 years for both sexes (Bell et al., 2005; McLaren, 1993). Because the body length range is relatively small, especially for males, and that variability in body size within age can be high (Bell et al., 2005; McLaren, 1993), concluding whether the effect of body size is driven by differences in growth rate or age may be challenging.

### 4.4 Boldness

We tested the effect of an individual's boldness on the seasonal habitat selection. Here, we assumed that our boldness index, that is, seals that showed the most muted responses to the toy cow, would take more risks in a foraging context (Dammhahn & Almeling, 2012). Thus, we first expected bolder individuals to select more strongly rich but risky ice‐covered habitats (i.e., the Antarctic shelf and the Oceanic Antarctic Zone), mainly in the winter. We found no conclusive evidence for this effect for females. As females tend to move northward with the marginal ice zone (Labrousse et al., 2015), this variability in their movement cannot be captured when defining habitats at the large scale. We thus hypothesize that differences in female risk taking may affect how they use the marginal ice zone, which requires to link the movement of the seals directly with the ice edge dynamics (Bailleul et al., 2007; Labrousse et al., 2015).

By contrast, we found some evidence for bolder males to select the Antarctic Shelf in the winter. This suggests that bolder males may be more prone to forage and risk being trapped in the ice pack (Labrousse et al., 2018). Several other studies have shown the increasing use of riskier habitats with boldness (Bonnot et al., 2018; Carrete & Tella, 2010; Holtmann et al., 2017). For example, shy female roe deer (*Capreolus capreolus*) used safer woodland areas more frequently, whereas bold individuals exploited rich open habitats (Bonnot et al., 2018). However, the evidence for positive correlation between boldness and habitat risk level remains equivocal, for example, bolder bank voles (*Myodes glareolus*) occupied microhabitats with more vegetation cover reducing predation risk compared to shyer individuals (Schirmer et al., 2019). These contrasting outcomes may emerge due to the nature of the ecological process generating the personality–habitat correlation which is not necessarily related to the benefit–cost trade‐off.

We consider the Kerguelen–Heard Plateau as a riskier habitat compared to other oceanic habitats due to higher levels of predation risk (van den Hoff & Morrice, 2008), competition (Hindell et al., 2011; O’Toole et al., 2017), and intra‐ and interannual variability (Pauthenet et al., 2018). We found that bolder females, but not males, increased the selection of the Kerguelen–Heard Plateau in the summer in contrast to winter. Females have been shown to specialize in foraging mainly in oceanic habitats (Bailleul, Authier, et al., 2010; Dragon et al., 2010; Guinet et al., 2014). A bloom in primary production occurs annually on the Kerguelen–Heard Plateau during the spring (Mongin et al., 2008). By the time the phytoplankton development reaches higher trophic levels, this habitat displays richer prey aggregations at the end of the spring and the beginning of the summer (Cotté et al., 2015). This could explain why bolder females select the Kerguelen–Heard Plateau in the summer as the increase in prey profitability may overcome the general costs, such as predation and competition with males. These results reinforce the context dependency of the foraging risk avoidance trade‐off with resource availability (Biro et al., 2003). Similarly, it was shown in seabird species that bolder individuals tend to use habitats closer to the colony compared to shyer ones presumably due to their higher competitive capacity (Krüger et al., 2019; Patrick & Weimerskirch, 2014).

The North Polar Front habitat, characterized by warmer surface water temperatures, was avoided by SESs of both sexes which is consistent with previous studies (Bailleul, Authier, et al., 2010; Dragon et al., 2010). Nonetheless, we found that the selection of this habitat increased with boldness in the winter, and this was especially evident in male seals. SESs were recorded to dive deeper in warmer waters to reach prey patches (Biuw et al., 2007; Guinet et al., 2014; McIntyre et al., 2011), such as the larger and energetically richer myctophids that typically occur in the warmer waters (Daneri & Carlini, 2002; Guinet et al., 2014; Slip, 1995). For a given prey catch level, individual SESs that foraged north of the subantarctic front increase their body condition faster than individuals that foraged in higher latitudes, revealing that they encounter larger or better quality prey items (Richard et al., 2016). Our results suggest that bolder individuals spend more time and energy in descent and ascent phases within dives to reach higher prey quality. This could be explained by individual differences in metabolic cost and life‐history productivity driven by the pace‐of‐life syndrome (Careau et al., 2008; Réale, Garant, et al., 2010). Boldness is usually positively correlated with growth rate or fecundity resulting in differences among individuals in energy requirements (Biro et al., 2014). Bolder individuals may thus target habitats with higher prey quality to make up for their higher energetic needs. From the novel object approach test, we interpreted the low response intensity of bolder individuals as a sign of low stress level resulting in lower metabolic costs (Careau et al., 2012). This may compensate for the extra energy expenditure bolder individuals spend when diving deeper in warmer waters.

Among the different eco‐evolutionary mechanisms explaining the link between individuals and their habitat (Edelaar & Bolnick, 2019), we can easily reject local adaptation or individual alteration of the environment in the Kerguelen SES situation. Individuals may thus either select habitats that best suit their phenotypes (i.e., matching habitat choice hypothesis) or the phenotypes of individuals may be shaped in response to environmental conditions (i.e., developmental plasticity hypothesis). The matching habitat choice hypothesis is the most plausible explanation as SESs are highly philopatric, which minimizes environmental differences among individuals when hauling out at the same site. However, we cannot reject the phenotypic plasticity hypothesis. Interannual differences in environmental conditions, for example, due to the Southern Annual Mode and the El Niño‐Southern Oscillation (Lovenduski & Gruber, 2005; Turner, 2004), at the first trip at sea may contribute to shape an individual's personality (Stamps & Groothuis, 2010). For example, in northern elephant seals, the variability in climate conditions mediates the composition of habitat fidelity strategies in the population (Abrahms et al., 2018).

The repeatability of the seal boldness score was found lower than the typical average value of ca. 0.37 (A. M. Bell et al., 2009). Although we tested the boldness of 76 individuals, we conducted repeated trials only on 13 females, which may be the cause of the low repeatability we recorded in addition to reducing the power of detecting correlations between boldness and habitat selection (Dingemanse & Dochtermann, 2013). Despite the low sample size, we found that boldness explained some of the individual variance in habitat selection. However, our results on the effect of boldness should be interpreted carefully until larger samples are available to more fully resolve the inter‐ and intraindividual variances (Niemelä & Dingemanse, 2018).

## 5 CONCLUSION

We show the complexity and importance of integrating several intrinsic factors (e.g., physiological, morphological, behavioral, and life‐history traits) into the same ecological framework to understand among‐individual variability in space use over time. We provided novel evidence that personality, in addition to sex and body size, explains partially the seasonal foraging habitat selection in SESs which may be driven by how individuals respond to environmental heterogeneity, for example, the landscape of fear or the energy landscape (Gallagher et al., 2017). Our findings provide a powerful link between some of the intrinsic variables associated with personality and extrinsic factors such as habitat structure which are essential aspects to comprehensively understand how animals use space and how this affects vital rates (i.e., survival and fecundity).

## CONFLICT OF INTEREST

None declared.

## AUTHOR CONTRIBUTIONS

**Hassen Allegue:** Conceptualization (lead); Data curation (supporting); Formal analysis (lead); Funding acquisition (supporting); Investigation (lead); Methodology (lead); Project administration (supporting); Validation (equal); Visualization (lead); Writing – original draft (lead); Writing – review & editing (equal). **Christophe Guinet:** Conceptualization (supporting); Funding acquisition (lead); Investigation (supporting); Methodology (supporting); Project administration (lead); Resources (lead); Supervision (equal); Validation (supporting); Writing – review & editing (equal). **Samantha C. Patrick:** Conceptualization (supporting); Formal analysis (supporting); Investigation (supporting); Methodology (supporting); Supervision (supporting); Validation (supporting); Writing – review & editing (equal). **Mark A. Hindell:** Funding acquisition (equal); Resources (equal); Validation (supporting); Writing – review & editing (equal). **Clive R. McMahon:** Funding acquisition (equal); Resources (equal); Validation (supporting); Writing – review & editing (equal). **Denis Réale:** Conceptualization (supporting); Formal analysis (supporting); Investigation (supporting); Methodology (supporting); Project administration (supporting); Resources (supporting); Supervision (equal); Validation (supporting); Visualization (supporting); Writing – review & editing (equal).

## Supporting information

Appendix S1

## Data Availability

The data from the CTD‐Satellite Relay Data Loggers are freely available through the International MEOP Consortium (http://www.meop.net/) and the Integrated Marine Observing System (IMOS, Australia; https://imos.org.au/). All datasets used in this study are provided on the Dryad Digital Repository https://doi.org/10.5061/dryad.g1jwstqs6
