# Supplementary material for: Sex, body size, and boldness shape the seasonal foraging habitat selection in southern elephant seals
Source: Ecol Evol. 2022 Jan 26;12(1):e8457. doi: 10.1002/ece3.8457 (PMC8796948; doi:10.1002/ece3.8457)
Supplement: Supplementary file 1 — Appendix S1 [file ECE3-12-e8457-s001.docx]

Appendix


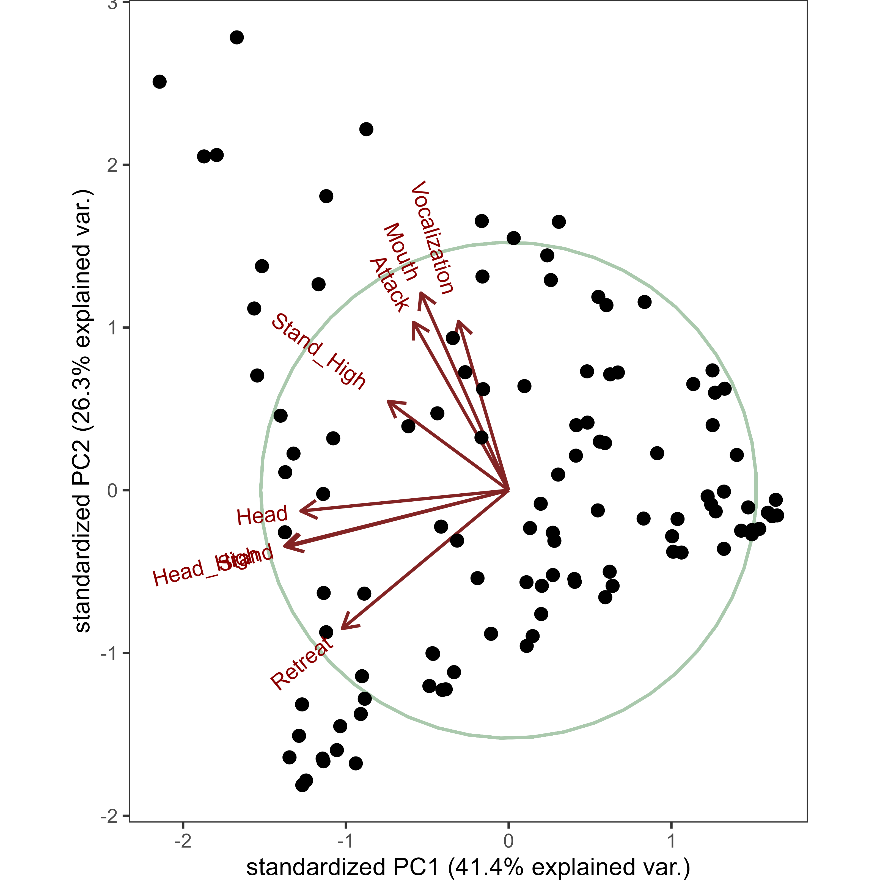


Fig S1: Biplot of the principal component analysis applied on the seal behavioural variables in response to the novel object approach test.


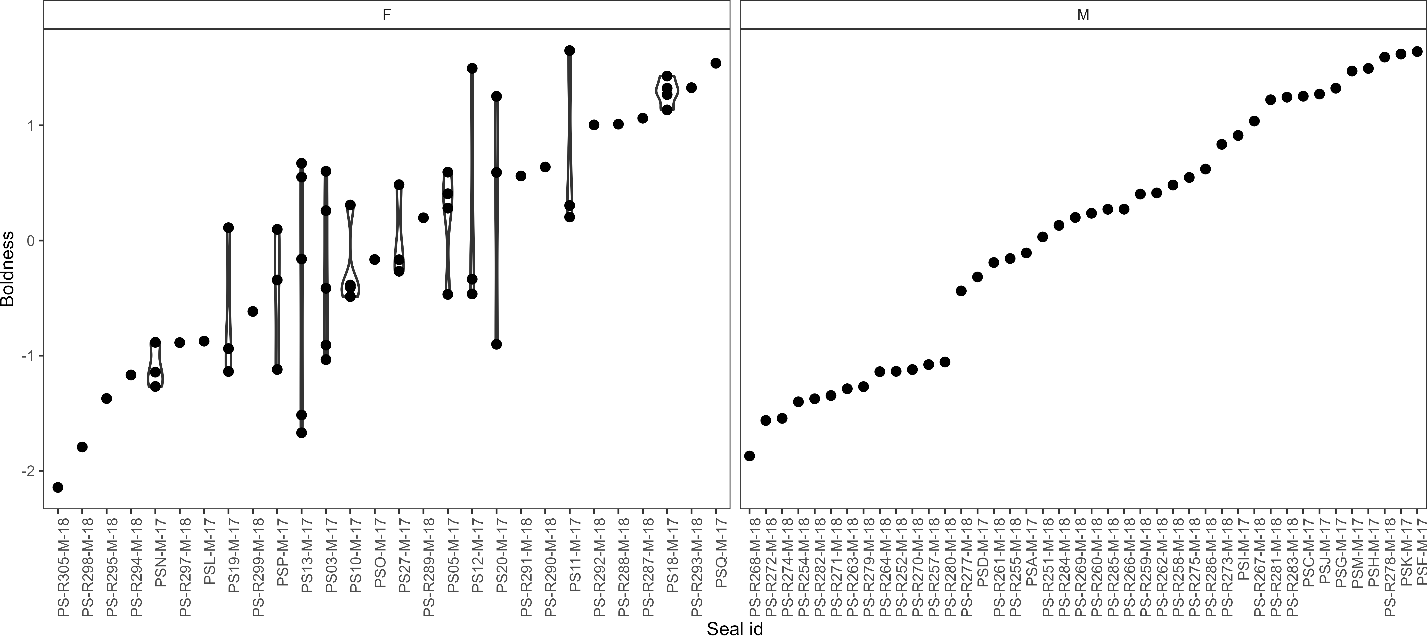


Fig S2: Normalized boldness scores of (F) female and (M) male seal individuals.


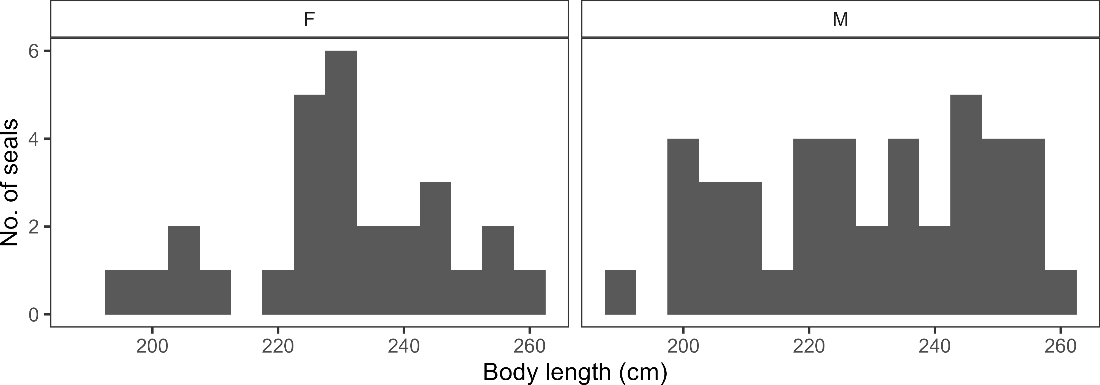


Fig S3: Histograms of body length (cm) of 28 female (F) and 42 subadult male (M) southern elephant seals.


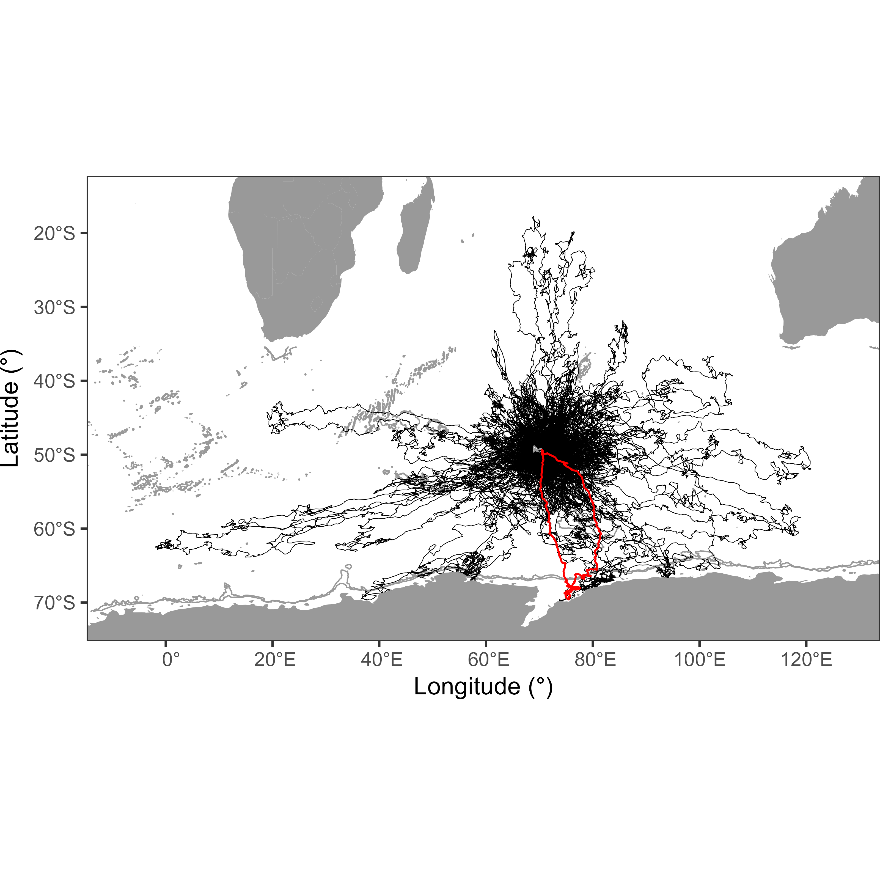


Fig S4: Example of a true seal track (red) and the randomly simulated pseudo-tracks (black) used to define habitat availability.


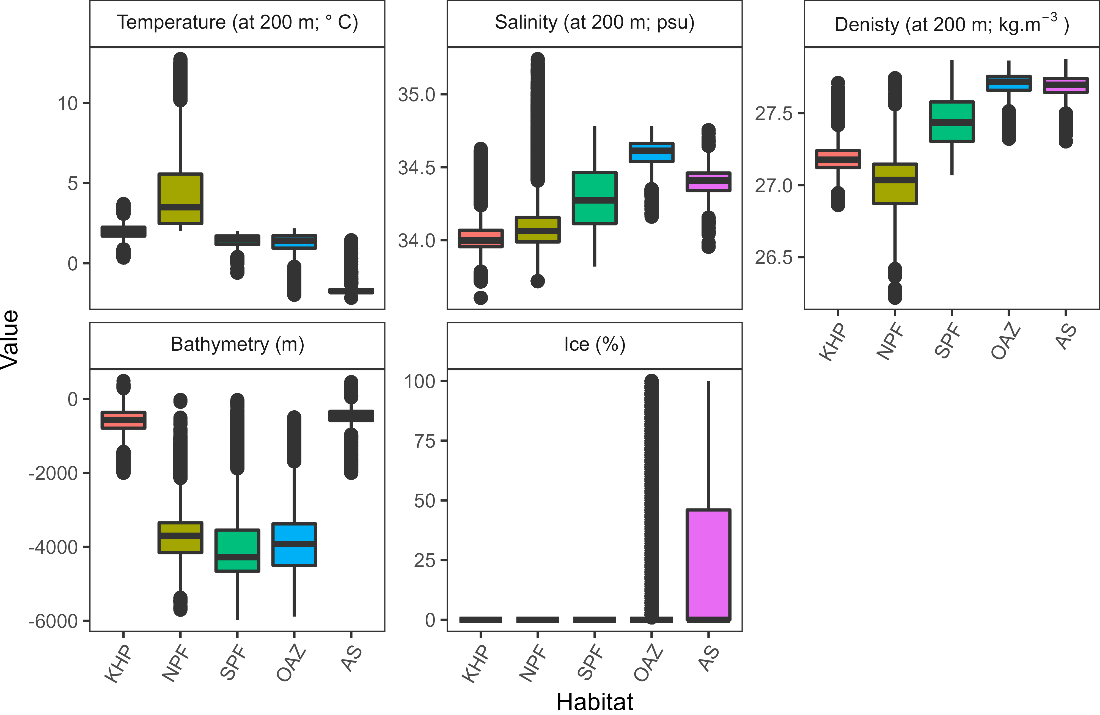


Fig S5: Boxplots of the temperature (°C), salinity (psu) and density (kg.m^-3^) at 200 m, bathymetry (m) and ice concentration (%) for each habitat category. Habitats are the Kerguelen-Heard plateau (KHP), the North of Polar Front (NPF), the South of Polar Front (SPF), The Oceanic Antarctic Zone (OAZ), and the Antarctic Shelf (AS).

Table S1: Output of the principal component analysis applied on the seal behavioural variables in response to the novel object approach test.

| Variables | Boldness  (PC1) |
| --- | --- |
| Head | -0.46 |
| Head High | -0.50 |
| Stand | -0.49 |
| Stand High | -0.27 |
| Mouth | -0.19 |
| Vocalization | -0.11 |
| Attack | -0.21 |
| Retreat | -0.37 |
| Variance explained | 41.4% |
| Eigen values | 3.3 |

Table S2: Model output of the first principal component axis (i.e., boldness) summarizing the seal behavioural responses to the novel object approach test. The parameter estimates or variances, the standard errors (SE), the t-statistics, and the 95% confidence intervals (CI) are presented. The position of the seals (i.e., straight or not), the number of times the seal was captured, and the year were selected as fixed effects. The seal identifier, the date (rounded to nearest hour value), and the tester identifier were added as random effects.

| Response variable | Term | Estimate | SE | t-statistic | CI |
| --- | --- | --- | --- | --- | --- |
| Boldness | *Fixed effects* |  |  |  |  |
|  | Intercept | 0.62 | 0.35 | 1.77 | [-0.07, 1.30] |
|  | Position [Not straight] | 0.63 | 0.18 | 3.47 | [0.27, 0.98] |
|  | No. capture | -0.27 | 0.10 | -2.67 | [-0.46, -0.07] |
|  | Year [2018] | -0.89 | 0.22 | -4.11 | [-1.31, -0.46] |
|  | *Random effects* |  |  |  |  |
|  | Id seal | 0.53 |  |  |  |
|  | Date | 0.00 |  |  |  |
|  | Id tester | 0.48 |  |  |  |
|  | Residuals | 0.70 |  |  |  |
|  | *Repeatability* | 0.28 | 0.15 |  | [0.03, 0.64] |
|  | *Marginal R^2^* | 0.18 |  |  |  |
|  | *Conditional R^2^* | 0.60 |  |  |  |
|  | *Sample size* | 76 |  |  |  |

Table S3: Among-trip variance (the diagonal)/correlation estimates between habitat pairs. Standard errors (SE) and 95% credible intervals [CI] are reported for each estimate. Bolded estimates do not include zero in the credible intervals. Habitats are the Kerguelen-Heard plateau (KHP), the North of the Polar Front (NPF), the South of the Polar Front (SPF), the Oceanic Antarctic Zone (OAZ), and the Antarctic Shelf (AS).

| Among-trip variance/correlation | | | | | |
| --- | --- | --- | --- | --- | --- |
|  | KHP | NPF | SPF | OAZ | AS |
| KHP | **1.20 (0.10)**  **[1.02, 1.40]** |  |  |  |  |
| NPF | **-0.72 (0.07)**  **[-0.85, -0.57]** | **2.99 (0.35)**  **[2.34, 3.73]** |  |  |  |
| SPF | **-0.88 (0.03)**  **[-0.93, -0.81]** | **0.70 (0.08)**  **[0.53, 0.84]** | **2.06 (0.18)**  **[1.73, 2.44]** |  |  |
| OAZ | **-0.69 (0.07)**  **[-0.82, -0.54]** | 0.20 (0.12)  [-0.04, 0.43] | **0.72 (0.07)**  **[0.57, 0.84]** | **4.05 (0.49)**  **[3.21, 5.14]** |  |
| AS | **-0.68 (0.08)**  **[-0.83, -0.50]** | 0.09 (0.13)  [-0.17, 0.36] | **0.52 (0.10)**  **[0.29, 0.70]** | **0.81 (0.07)**  **[0.66, 0.91]** | **4.45 (0.60)**  **[3.41, 5.77]** |
